# Haemostatic and thrombo-embolic complications in pregnant women with COVID-19: a systematic review and critical analysis

**DOI:** 10.1186/s12884-021-03568-0

**Published:** 2021-02-05

**Authors:** Juliette Servante, Gill Swallow, Jim G. Thornton, Bethan Myers, Sandhya Munireddy, A. Kinga Malinowski, Maha Othman, Wentao Li, Keelin O’Donoghue, Kate F. Walker

**Affiliations:** 1 Department of Obstetrics and Gynaecology, Nottingham University Hospitals NHS Trust, Nottingham, UK; 2 Department of Haematology, Nottingham University Hospitals NHS Trust, Nottingham, UK; 3 Division of Child Health, Obstetrics and Gynaecology, School of Medicine, University of Nottingham, Nottingham, UK; 4 Department of Haematology, University Hospitals of Leicester, Leicester, UK; 5 Division of Maternal-Fetal Medicine, Department of Obstetrics and Gynaecology, Mount Sinai Hospital, University of Toronto, Toronto, Ontario Canada; 6 Department of Biomedical and Molecular Sciences, School of Medicine, Queen’s University Kingston, Kingston, Ontario Canada; 7 School of Baccalaureate Nursing, St Lawrence College, Kingston, Ontario Canada; 8 Department of Obstetrics and Gynaecology, Monash University, Clayton, Australia; 9 The Irish Centre for Maternal and Child Health, University College Cork, Cork University Maternity Hospital, Cork, Ireland

**Keywords:** COVID-19, SARS-CoV-2, Pregnancy, Birth, Venous thrombosis, Arterial thrombosis, Coagulopathy, Disseminated intravascular coagulopathy, Haematological complications

## Abstract

**Background:**

As pregnancy is a physiological prothrombotic state, pregnant women may be at increased risk of developing coagulopathic and/or thromboembolic complications associated with COVID-19.

**Methods:**

Two biomedical databases were searched between September 2019 and June 2020 for case reports and series of pregnant women with a diagnosis of COVID-19 based either on a positive swab or high clinical suspicion where no swab had been performed. Additional registry cases known to the authors were included. Steps were taken to minimise duplicate patients. Information on coagulopathy based on abnormal coagulation test results or clinical evidence of disseminated intravascular coagulation (DIC), and on arterial or venous thrombosis, were extracted using a standard form. If available, detailed laboratory results and information on maternal outcomes were analysed.

**Results:**

One thousand sixty-three women met the inclusion criteria, of which three (0.28, 95% CI 0.0 to 0.6) had arterial and/or venous thrombosis, seven (0.66, 95% CI 0.17 to 1.1) had DIC, and a further three (0.28, 95% CI 0.0 to 0.6) had coagulopathy without meeting the definition of DIC. Five hundred and thirty-seven women (56%) had been reported as having given birth and 426 (40%) as having an ongoing pregnancy. There were 17 (1.6, 95% CI 0.85 to 2.3) maternal deaths in which DIC was reported as a factor in two.

**Conclusions:**

Our data suggests that coagulopathy and thromboembolism are both increased in pregnancies affected by COVID-19. Detection of the former may be useful in the identification of women at risk of deterioration.

**Supplementary Information:**

The online version contains supplementary material available at 10.1186/s12884-021-03568-0.

## Background

Outside pregnancy severe COVID-19 is prothrombotic and proinflammatory, and the presence of coagulopathy is associated with a poorer prognosis; 71% of patients who die have disseminated intravascular coagulopathy (DIC) as defined by the International Society on Thrombosis and Haemostasis (ISTH) criteria compared with 0.6% among survivors [1].

In the non-pregnant population, severe COVID-19 coagulopathy is characterised by a significantly elevated D-dimer concentration. Elevated D-dimers/fibrin degradation products are also seen in DIC as diagnosed according to the ISTH criteria [2, 3] and the pregnancy-specific DIC scoring system which has been developed to account for the relevant physiological adaptations [4]. However, unlike coagulopathy associated with other underlying causes, COVID-19 is less commonly associated with prolongation of prothrombin time (PT) and activate partial thromboplastin time (APTT) or thrombocytopenia [5, 6]. Fibrinogen appears to be at least initially well preserved although there have been reports of low fibrinogen, particularly in non-survivors [1, 7, 8].

Accumulating data demonstrate increased risk of thromboembolism in COVID-19, predominantly in the most severe intensive care unit (ICU) cases [9–12]. Middledorp et al. found a 25% incidence at 7 days, rising to 48% at 14 days in ICU patients [9]. Similarly, Cui et al. demonstrated that 20/81 (25%) of patients admitted to ICU developed thromboembolic complications, of which 8 died [10].

As pregnancy is already a physiologically hypercoagulable state, it seems likely that affected pregnant women would be at especially high risk of these complications. Current advice from the RCOG recommends that all pregnant women admitted with confirmed or suspected COVID-19 receive prophylactic low molecular weight heparin (LMWH), unless birth is expected within 12 h, and continue this for 10 days following discharge [13]. Risk factors for thromboembolic complications in pregnancy are well documented.

Although the number of pregnant women with COVID-19 included in scientific reports as of 6th July 2020 stands at 6742 [14], many of these reports include the same or overlapping cases [15]. Potential duplicate publication is particularly challenging for reports from Wuhan, China; a city of 12 million people with 50 hospitals, 19 of which have reported cases of COVID-19 in pregnancy, and many of which have multiple names in translation [16]*.* In the West, hospitals and registries similarly often cite the same cases. Here, we have removed potentially duplicate reports in a conservative manner: when in doubt data were excluded.

In this systematic review, we aimed to determine two estimates:

1. The rate of arterial or venous thrombosis in pregnant women with confirmed or suspected COVID-19
2. The rate of acquired coagulopathy in pregnant women with confirmed or suspected COVID-19

## Methods

Case reports and series of confirmed or suspected maternal COVID-19 in pregnancy were identified according to the methodology used by Walker et al. [17].

### Criteria for potentially eligible studies

Studies were eligible for inclusion if they were case reports or case series, of pregnant women with confirmed COVID-19 infection and where the outcome of the pregnancy (either ongoing or delivered) was reported. There was no language restriction. We only included cases where either the mother had confirmed COVID-19 based on a positive swab, or a high clinical suspicion of COVID-19 where a swab had not been taken e.g. symptoms and radiographic evidence in an area of high COVID-19 prevalence.

### Search strategy

We identified all scientific case reports and case series of confirmed or suspected maternal COVID-19 in pregnancy. The basis of the list was a curated list kept by the senior author (JGT) on his personal blog since March 22nd. This is a curated list of primary sources based on a daily PubMed search supplemented by alerts from colleagues on social media. After April 8th this list was supplemented by formal daily searches by KO and KFW.

The search was undertaken between 8th April to May 2020 through the following electronic bibliographic databases (Medline, Embase and Maternity and Infant Care Database) and citation tracking on relevant studies. The search terms associated with COVID-19 used in bibliographic databases were adapted in database-specific filters. The searches were re-run just before the final analyses and further studies retrieved for inclusion. The date of the last search was 05/06/2020. The search strategy is shown in Appendix 1. The dataset is available at: https://ripe-tomato.org/2020/05/15/covid-19-in-pregnancy-101-onwards/.

### Selection of studies

Titles and abstracts identified by the search strategy were assessed for inclusion by two reviewers (KW, KO). If there was disagreement about whether a report should be included, full text was obtained for that report.

For all potentially eligible studies full text copies were sought, and independently assessed for inclusion by two reviewers (KW, KO). Disagreements were resolved by discussion, and if agreement could not be reached the study was independently assessed by a third reviewer (JGT).

### Data extraction and data entry

Data on study quality and content were extracted onto an Excel spread sheet, and checked (KW, JGT). Where data was missing, the first author of the paper was contacted by email (*n* = 4). Data was collected on maternal outcomes.

### Data analysis

**Fig. 1:**
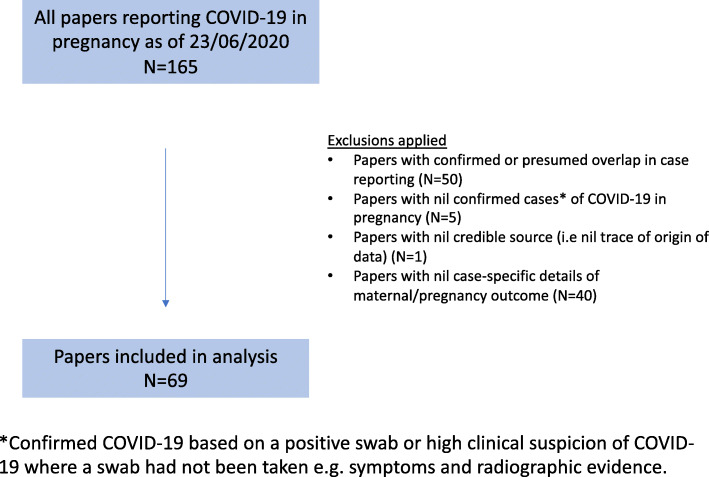
Flow chart of papers included in analysis. Papers were identified between 08/02/20 and 05/06/20 using methodology described by Walker et al. The original dataset is available at https://ripe-tomato.org/2020/05/15/covid-19-in-pregnancy-101-onwards/. Exclusion criteria were applied, and 69 papers were included in the final analysis

One-hundred-sixty-five papers were identified according to this methodology and 69 papers met inclusion criteria (see Fig. 1). Additional cases known to the authors were added from registries including the UK Obstetric Surveillance System (UKOSS) database, the East Midlands Research group (a group recently formed for the investigation of non-malignant haematological changes in pregnancy) and from the International Society on Thrombosis and Haemostasis’ Pregnancy and COVID-19-Associated Coagulopathy (COV-PREG-COAG) Registry.

Coagulopathy events were recorded as stated by the authors. If haematological results were given, the DIC in pregnancy score was calculated, based on the prothrombin time, platelet count and fibrinogen levels. This scoring system has shown a sensitivity of 88% and a specificity of 96% for the diagnosis of DIC in pregnancy [4].

Few papers specifically stated negative findings for coagulopathy or thrombosis. Cases were therefore considered negative for these events if it was specified that there were no complications during the observed clinical course, or if patients were stated to have recovered/be recovering, or discharged without mention of coagulopathy or thrombosis.

Characteristics of each study were described and tabulated. Confidence intervals for the outcomes given were calculated using software available at: https://epitools.ausvet.com.au/ciproportion.

## Results

Details for 1063 women with COVID-19 in pregnancy have been reported, where maternal outcomes were provided. Of these, three (0.28, 95% CI 0.0 to 0.6)) have had thromboembolic disease, seven (0.66, 95% CI 0.17 to 1.1) have been diagnosed with DIC, with another three (0.28, 95% CI 0.0 to 0.6)) noted to have a coagulopathy. Five hundred and thirty-seven (56%) have been reported as recovered/recovering and having given birth and 426 (40%) have been reported as recovered/recovering with ongoing pregnancy (Table 1). In addition, Pereira et al described 2/60 patients with deep vein thrombosis (DVT); however, this report was discounted from the above totals (and Table 1) due to lack of reported pregnancy outcomes [7].

**Table 1:** Summary of all reported cases with haemostatic complications, coagulopathies or DIC in pregnant women with confirmed COVID-19 infection

Location	Source (Study number as per ripe-tomato.org or database)	Pregnant women with confirmed COVID-19 infection with outcomes reported	Women who required critical care. N/A not available	Women who delivered, presumed healthy^1^	Women with ongoing pregnancies, presumed healthy^1^	Venous thrombotic events	Arterial thrombotic events	Disseminated intravascular coagulation (DIC) events
China								
Zhongnan Hospital of Wuhan University	1	9	0	9	0	0	0	0
Union Hospital, Tongji Medical College, Huazhong University of Science and Technology	2a	3	0	3	0	0	0	0
The first Affiliated Hospital, College of Medicine, Zhejiang University	4	1	0	1	0	0	0	0
Union Hospital, Tongji Medical College, Huazhong University of Science and Technology	6	15	0	11	4	0	0	0
Qingdao Women and Children’s Hospital, Qingdao	7	1	0	0	1	0	0	0
Tongji Hospital, Tongji Medical College, Huazhong University of Science and Technology	15	7	0	7	0	0	0	0
Affiliated Infectious Hospital of Soochow University, Suzhou	19	1	1	1	0	0	0	0
Maternal and Child Hospital of Hubei Province	30	34	0	34	0	0	0	0
Beijing YouAn Hospital, Capital Medical University	34	1	0	1	0	0	0	0
Renmin Hospital of Wuhan University	36	17^3a^	N/A	14	0	0	0	0
Renmin Hospital of Wuhan University	37	3	0	3	0	0	0	0
No 2 People’s Hospital of Hefei City Affiliated to Anhui Medical University	62	1	0	1	0	0	0	0
Central Hospital of Wuhan	73	28^4e^	N/A	22	2	0	0	0
Xiaolan People’s Hospital of Zhongshan, Guandong	81	1	1	1	0	0	0	1
USA								
MedStar Washington Hospital Center, DC	21	1	0	1	0	0	0	0
Newark Beth Israel Medical Centre, New Jersey	28	2^a,b^	N/A	0	0	0	0	0
Saint Barnabas Medical Center, Livingston, New Jersey	111	1	1	1	0	0	0	0
Morristown medical centre, St Peter’s University Medical Centre, New Jersey	159	3	3	0	3	0*	0	0
“Network’s 2 largest hospitals” in New Jersey: Likely Hackensack University Medical Centre, Rutgers New Jersey Medical School Newark, Seton Hall University Nutley, Jersey Shore University Medical Centre, Neptune,	149	8	8	7	1	0	0	0
Good Samaritan Hospital, Cincinnati, Ohio	50	1^b^	1	0	0	0	0	0**
Lexington Medical Centre, West Columbia, South Carolina	60	1	0	0	1	0	0	0
Hospital of the University of Pennsylvania	65	5^b^	5	3	1	1	0	0
Washington University in St Louis, Missouri	69	1	1	1	0	0	0	0
Beaumont Hospital Dearborn, Dearborn, Michigan	123	16	0	10	6	0	0	0
Henry Ford Hospital Department of Obstetrics and Gynecology, Detroit, Michigan (distinct case from 123)	87	1	1	0	1	0	0*	0
University of California, San Francisco, California	89	1	1	1	0	0	0	0
Stanford University Hospital, California	115	1	0	1	0	0	0	0
New York University, Winthrop hospital, Langone health	91	1	1	1	0	0	0	0
New York University, Langone Health (distinct case)	98	1^c^	0	0	0	0	0	0
Weil Cornell Medicine, New York**	99	20^h^	0	19	0	0	0	0
Norwell Group, New York	118 162	13^2g,a^	13	5	5	0	0	0**
New York University, Langone health	152	2	2	2	0	0	0	0
Six hospital systems in Washington state	102	46	1	8	38	0	0	0
University of Tennessee Health Science Center, Nashville, United States	112	1	1	0	1	0	0	0
Yale School of Medicine	155	1^e^	N/A	0	0	0	0	1
Advocate Good Samaritan Hospital, Illinois, United States^	COV-PREG-COAG	1	0	1	0	0	0	0
St Joseph Hospital, Denver	156	1	0	1	0	0	0	0
Canada								
Mount Sinai Hospital, Toronto	48	1	0	1	0	0	0	1
Honduras								
Hospital Escuela de Tegucigalpa	18	1	0	1	0	0	0	0
Sweden								
Southern General Hospital, Stockholm	20	1	0	1	0	0	0	0
France								
Antoine Beclere Hospital, Clamart	48	1	N/A	1	0	0	0	1
Hospitaux Universitaires de Strasbourg	161	54^a,b,d^	5	20	31	0	0	0
Canary Islands								
Hospitalario Universitario Insular Materno Infantil, Gran Canaria	53	1	1	1	0	0	0	0
Italy								
Fondazione Policlinico Universitario A. Gemelli IRCCS, Rome, Italy	76	7^c^	N/A	4	2	0	0	0
12 Italian hospitals (non-overlapping with others in table)	117	26	14	6	20	0	0	0
Parma Hospital, Italy	109	4	N/A	4	0	0	0	0
6 hospitals of Azienda USL 62 “Toscana Nord Ovest” [ATNO] (Tuscany), and Gaslini Children’s Hospital (Genoa, Liguria)	133	3	0	3	0	0	0	0
UK								
Portland Hospital London	82	8	0	8	0	0	0	0
East Midlands Research group (University Hospitals of Leicester and Nottingham University Hospitals)	East Midlands Research Group	30^b,g^	2	21	7	0	0	1
UK (Nationwide)- UKOSS database with case information as per paper 107.	UKOSS + 107	427^5g^	41	261	161	1	1	0
Belgium								
Cliniques Universitaires, St Luc, Brussels,	100	1	0	1	0	0	0	0
4 Obstetric units in North East Flanders	128	13	0	13	0	0	0	0
Portugal								
Hospital Pedro Hispano	105	12	0	10	2	0	0	0
Porto (distinct case)	94	1	0	1	0	0	0	0
Portugal (distinct case)	74	1	0	1	0	0	0	0
Netherlands								
Netherlands COVID-19 registry	141	176^d,g,i^	7	49	124	1^g^	0	0
Germany								
Ulm university	127	2	0	2	0	0	0	0
Spain								
Jaen	158	4	0	0	4	0	0	0
Barcelona	140	8	8	4	4	0	0	0
South Korea								
Daegu Fatimal Hospital	22	1	0	1	0	0	0	0
Japan								
Keio University Hospital, Tokyo	144	2	0	0	2	0	0	0
Turkey								
Ankara University Faculty of Medicine,	31	1^b^	1	0	0	0	0	0
Sehit Prof Dr. Ilhan Varank Sancaktepe Training and Research Hospital, Istanbul	146	8^c^	1	2	5	0	0	0
Necmettin Erkbakan University, Konya	145	1	0	1	0	0	0	0
Jordan								
Jordan	153	1	0	1	0	0	0	0
Australia								
Gold Coast University Hospital	45	1	0	1	0	0	0	0
India								
Designated Covid Hospital	58	1	0	1	0	0	0	0
Iran								
Tehran/Rasht/Qom/Zanjan	67	9^7g,b^	9	1	0	0	0	1**
Imam Khomeini Hospital, Sari, Iran	70	1^g^	1	0	0	0	0	0
Imam Reza Hospital of Tabriz, Iran	101	1^b^	1	0	0	0	0	1
Thailand								
Thailand (reported by ministry of public health)	110	1^f^	0	0	0	0	0	0
Russia								
Russian Federation, Private Center^	COV-PREG-COAG	1	0	1	0	0	0	0
UAE								
Al Ain Hospital, United Arab Emirates^	COV-PREG-COAG	1	0	1	0	0	0	0
Location		Pregnant women with confirmed COVID-19 infection with outcomes reported	Women who required critical care. N/A not available	Women who delivered, presumed healthy^1^	Women with ongoing pregnancies, presumed healthy^1^	Venous thrombotic events	Arterial thrombotic events	Disseminated intravascular coagulation (DIC) events
Total		**1063**	**132/1033**	**593**	**426**	**3**	**1**	**7**

a-remains inpatient (6), b-remains inpatient- stated to be on ITU/ventilator (8), c-Pregnancy loss before 24 weeks (3), d-pregnancy loss (gestation not stated) (2), e-termination before 24 weeks (due to COVID-19) (5), f- termination before 24 weeks (other reason) (1), g-patient died (17), h-readmission with nil further details (1), i-molar pregnancy (1)

*line thrombosis noted (see Table 2)

**Additional coagulopathy noted (see Table 3)

*** Isolated abnormal coagulation parameters- not specified further

^1^Few papers specifically stated negative findings for coagulopathy or thrombosis. Cases were therefore considered negative for these events if it was stated that there were no complications during the observed clinical course, or if patients were stated to have recovered/be recovering, or discharged without mention of coagulopathy or thrombosis

Tables 2 and 3 provide summaries of reported cases of thrombosis and coagulopathy respectively, in pregnant women confirmed or highly-suspected to have COVID-19 as taken from Table 1.

**Table 2:** Summary of reported cases with venous and arterial thrombotic events in pregnant women with confirmed COVID-19 infection

Case	Study number	Number requiring critical care	Number of maternal deaths	Type of arterial thrombotic events	Type of venous thrombotic events 1 = inferior vena cava 2 = pulmonary embolism 3 = DVT	Number symptomatic	Diagnosis of event made antenatally (1) or postnatally (2)	Number receiving thromboprophylaxis prior to VTE event	If thromboprophylaxis reported, what type and what dose?	D-dimer measurement (micrograms/ml normal = < 0.5)	Risk factors: PET = 1, smoker = 2, FHx VTE = 3, Age > 35 = 4, IVF = 5, twins = 6, parity > 3 = 7, BMI > 30 = 8
1	65	1	0	0	1		1	1	“therapeutic anticoagulation”	Not given	7
2	87	1	0	0^a^	0		1	1	enoxaparin 40 mg subcutaneously daily. BMI 41.5	0.57–2.82	4,8
3	UKOSS (107)	1	1	Basilar artery thrombosis	2	1 “Deteriorating respiratory function”	2	1	Enoxaparin (prophylactic dose)	Not given	8,
4	141		1	0	2						
5	159	1	0	0	0^b^	0	1	1	Lovenox 40 mg daily	Not given	4,5

^a^ Arterial line required replacement multiple times due to thrombosis despite VTE prophylaxis”

^b^ Patient was undergoing dialysis via central venous line catheter. “Despite the thromboprophylaxis, the blood repeatedly coagulated in the dialysis machine. Thus, the patient was started on a continuous heparin drip”

^1^Few papers specifically stated negative findings for coagulopathy or thrombosis. Cases were therefore considered negative for these events if it was stated that there were no complications during the observed clinical course, or if patients were stated to have recovered/be recovering, or discharged without mention of coagulopathy or thrombosis

**Table 3:** Summary of reported cases of disseminated intravascular coagulation (DIC) or coagulopathy in pregnant women with confirmed COVID-19

		Case 1	Case 2	Case 3	Case 4	Case 5	Case 6	Case 7	Case 8	Case 9	Case 10
Study number		**48 (Canada)**	**48**	**67**	***67***	**50**	**118**	**NUH/UHL**	**155**	**101**	**81**
Classification of coagulopathy		**DIC in pregnancy score 27**	**DIC in pregnancy score 27**	**Authors stated DIC**	**Authors stated coagulopathy**	**Authors state mild coagulopathy. DIC in pregnancy score 27**	**Authors stated coagulopathy**	**Authors stated DIC**	**Authors stated DIC**	**DIC in pregnancy score 27**	**Authors stated DIC**
Maternal outcome		**Recovered**	**Recovered**	**Died**	**Remains in Hospital**	**Remains on ICU**	**Died**	**Died**	**Recovered** **(after termination of pregnancy)**	**Remains in hospital**	**Recovered**
Haematological indices	**Platelets (minimum and maximum if multiple values reported)**	**82**	**54**	**122–188**	**122–170**	**114**	**40–119**	**57**	**33–94**	**required “10 injections of platelets)**	**57**
	**APTT (normal range)**	**41 (18.5–29.9)**	**60 (28.0–41.9)**			**35.1**	**PTT 30.1–30.6**	**49.3 (24–33)**	**PTT 44.6–27.7**	**PTT 36**	
	**Prothrombin Time**					**20.2**	**10.6–10.9**	**23.9**	**12.7**	**16**	
	**INR**	**1.0**	**1.1**			**1.7**	**0.94–0.97**	**1.8**			
	**Fibrinogen (g/L)** ***Normal 2.48–5.06 (3rd trimes ster)***	**2.2**	**0.8**					**1.1**	**Mg/dL < 60–275**		
	**D Dimer (mg/L)** ***normal 0.13–1.7***	**25.79**	**> 20**				**6.5**	**19.06**	**> 33.89**		
	**Minimum ISTH Pregnancy DIC Score with available values**	**27**	**27**			**27**		**N/A (postpartum)**	**26**	**27**	
	**Minimum DIC score (ISTH)**	**4**	**5**				**2**	**6**	**6**		

^1^Few papers specifically stated negative findings for coagulopathy or thrombosis. Cases were therefore considered negative for these events if it was stated that there were no complications during the observed clinical course, or if patients were stated to have recovered/be recovering, or discharged without mention of coagulopathy or thrombosis

Of 1063 pregnant women included in our current study, there were 17 deaths (1.6, 95% CI 0.85 to 2.3). DIC was reported in two of these cases (12%). We also noted a higher incidence of thrombotic events in non-survivors, with pulmonary embolism occurring in two cases (distinct to the cases of DIC) and concurrent basilar artery thrombosis in one case. One hundred and thirty two/1033 (13.0%) women with COVID-19 in this study required admission to ICU.

Platelet levels and D-dimers were reported in several cases where haematological results did not meet the criteria for DIC and patients had not been stated to have a coagulopathy. In addition to cases noted to have a coagulopathy, D-dimer was noted to be raised (as reported by authors or above 0.5 mg/l) in 31 of 38 cases [18–33], and from the COV-PREG-COAG Registry] where a value was reported or commented on. Platelets were low (as reported by authors or < 100) in 15 of 102 cases where a value was reported or commented on [18, 19, 21, 23, 24, 27–30, 33–40], also cases from the COV-PREG-COAG Registry] (see Appendix 2).

## Discussion

### Statement of principle findings

Haemostatic and thromboembolic complications have been reported in 0.98 and 0.28% of pregnant women with COVID-19 infection respectively**.** The absolute risk of thromboembolic complications in pregnant women without COVID-19 is 0.1% [41]. Estimates of the incidence of DIC in pregnant women range between 0.03 to 0.35% [42]. Our findings suggest that the risk of haemostatic and thromboembolic complications are higher in pregnant women with COVID-19 infection than in pregnant women without COVID-19 infection.

### Strengths and limitations

Our review is the largest reported to date, even following removal of potential duplicates. The precision of our estimates is therefore greater.

Many primary studies were case reports or hospital-based series, which are at risk of bias towards cases or findings of interest, resulting in potential overestimation of complications. On the other hand, few papers specifically stated that there were no haemostatic complications in each case. Our assumption that this means an absence of complications may result in an underestimate, as theoretically complications may have been present, but not reported.

The DIC score used to identify cases from laboratory findings is a composite of prothrombin time, platelet counts and fibrinogen levels [4]. However, coagulopathy in COVID-19 is associated with a modest change in these parameters [5], meaning that the DIC score alone may be less accurate as a measure of COVID-19 coagulopathy in pregnancy. In addition, many authors did not report fibrinogen levels or prothrombin time, which will have falsely lowered our rate estimate of coagulopathy. D-dimer, like C-reactive protein (CRP), is an acute phase reactant, which can be elevated in trauma or any inflammatory condition. Elevated D-dimer levels are difficult to interpret, as the etiology of their rise can be multifactorial. D-dimer elevations can occur during an uncomplicated pregnancy, though typically they are not as pronounced as in some of the cases in this study, where the values were reported. Pneumonia as well has been associated with high D-dimer levels, as have thromboembolic events. As reported in Pereira et al*,* pregnant women who were classified as having severe clinical features of pneumonia in COVID-19 had higher D-dimer and CRP [7]. On the other hand, significant elevations of D-dimer were also noted in two reported cases of COVID-19 associated coagulopathy in pregnancy, neither of which were complicated by pneumonia or significant respiratory compromise [42]. While lack of standardisation of D-dimer thresholds in pregnancy renders interpretation challenging, in these two cases D-dimer levels were grossly elevated, at 17- and 12- fold the upper limit of normal [42].

The efficacy of D-dimer in the diagnosis of pulmonary embolism (PE) in pregnancy has been investigated, with conflicting results. The DiPEP (diagnosis of PE in pregnancy) group concluded, using D-dimer measurement by ELISA (counted as negative if < 400 ng/ml) and using Innovance technology (reference range 1–1.3 mg/L), that D-Dimer was not useful for the diagnosis of PE in the context of pregnancy [43]. However, Van der Pol et al. reported that D-dimer measurement could be used in order to rule out PE in this group [44], using a cut of value of > 1000 ng/ml if nil clinical criteria were met, or < 500 ng/ml where wither there were clinical signs of either deep vein thrombosis; haemoptysis or where PE was the most likely diagnosis. Thus, the potential prognostic value of D-dimer in pregnancy in the setting of COVID-19 cannot be dismissed outright and deserves further investigation. Additionally, other tools for assessing hypercoagulability or other forms of coagulopathy such as Thromboelastography™ /Thromboelastometry™ are worth evaluating. An ISTH review and recommendation for the use of these technologies in obstetrics has recently been published [45].

### Comparison with previous studies

Sentilhes [33] found no cases of thromboembolic disease or thrombocytopenia among 54 pregnant women with COVID-19 including five women who were admitted to ICU in Strasbourg. Guan [46] reported one case of DIC among 1099 cases of laboratory confirmed COVID-19 in non-pregnant patients of all ages (0.1% of cases). Tang [1] noted a higher incidence of coagulopathy in non-survivors which is in keeping with our findings. Whilst uncommon in pregnant women with COVID-19, our data suggests that the identification of haemostatic and coagulopathic changes may have value in the identification of women at risk of deterioration.

## Conclusion

### Implications for clinical practice

Our findings suggest that haematological complications are more commonly observed in pregnant women with COVID-19 infection (1.26%) than in pregnant women without (0.45%) and support the current advice from the RCOG recommending that all pregnant women admitted with confirmed or suspected COVID-19 receive prophylactic low molecular weight heparin (LMWH), unless birth is expected within 12 h, and continue this for 10 days following discharge.

Despite findings of elevated D-dimer in patients who have tested positive for COVID-19 outside of pregnancy, the occurrence of DIC and thrombotic events is infrequently reported [6]. We have found this to also be the case where COVID-19 is described in pregnancy; perhaps in part due the resultant coagulopathy being distinct from DIC and/or secondary to a lack of standardised cut off values for coagulation parameters for the diagnosis of coagulopathy in COVID-19 in the context of pregnancy. Nonetheless, identification of haemostatic and thrombotic complications may still be of clinical importance in recognizing pregnant patients who are at a higher risk of mortality from COVID-19.

To diagnose coagulopathy in a pregnant woman with COVID-19, we would recommend checking a full blood count, D dimer/fibrin degradation products (FDP), clotting screen and fibrinogen and using these parameters to calculate the pregnancy related DIC score. These parameters are useful if the woman needs delivery and can guide blood product support. Othman et al provide practical suggestions on interpretation of these laboratory parameters based on expert consensus [8].

Despite findings of DIC, there is no evidence that correcting abnormal coagulation parameters in patients who are not actively bleeding is beneficial. This advice covers all patients with COVID associated DIC. The only difference for pregnant women would be if they required delivery. Do not use tranexamic acid; recovery from DIC is dependent on endogenous fibrinolysis to break down the disseminated thrombi. This process is inhibited by tranexamic acid, an anti-fibrinolytic drug. If there is bleeding associated with DIC give blood product replacement.

Given the increased chances of thrombosis in a normal pregnancy there needs to be a high index of suspicion of VTE in this patient group if they also have COVID-19. One cannot rely on the D dimer to determine chances of VTE; you should not do that anyway even without COVID but in COVID it is likely to be much higher. If the woman is near to delivery, then the coagulation parameters and platelet count will have potential implications for delivery and guidance from a haematologist would be appropriate on an individual patient basis.

Investigation and management for suspected thrombosis should be the same as non-COVID pregnant woman.

### Implications for research

Continued collection of data on specific parameters of thrombosis and haemostasis from pregnant women affected by COVID-19 is necessary to further elucidate the incidence, prognostic value, and implications of coagulopathy, and thromboembolism in pregnancy.

More detailed investigation of coagulation abnormalities may also be useful. These could include studies such as specialised factor assays (taking into account the normal haemostatic changes that occur in pregnancy).

Determination of specific cut-off values of aberrant haemostatic parameters associated with adverse outcomes in pregnancy is needed. Given the rarity of the condition, even in the face of a global pandemic, and in absence of systematic studies or until data from randomised control trials become available, international registries can be of immense value in achieving this aim. The International Society on Thrombosis and Haemostasis has developed the Pregnancy and COVID-19-Associated Coagulopathy (COV-PREG-COAG) Registry, precisely to fulfil this aim. Participation in the Registry is open to health care providers worldwide and can be accessed at: https://redcap.isth.org/surveys/?s=4JPX9W98RH.

## Supplementary Information

**Additional file 1.**

**Additional file 2: Appendix 2.** D-dimer levels and platelet levels where reported for cases of COVID-19 in pregnancy.

## Data Availability

The datasets used and/or analysed during the current study available in the supplementary files.

## Abbreviations

APTT: Activate partial thromboplastin time
CRP: C-reactive protein
DIC: Disseminated intravascular coagulation
DVT: Deep vein thrombosis
ICU: Intensive care unit
ISTH: International Society on Thrombosis and Haemostasis
LMWH: Low molecular weight heparin
PE: Pulmonary embolism
PT: Prothrombin time
RCOG: Royal College of Obstetrics and Gynaecology
UKOSS: UK Obstetric Surveillance System
