# Supplementary material for: Haemostatic and thrombo-embolic complications in pregnant women with COVID-19: a systematic review and critical analysis
Source: BMC Pregnancy Childbirth. 2021 Feb 5;21:108. doi: 10.1186/s12884-021-03568-0 (PMC7863033; doi:10.1186/s12884-021-03568-0)
Supplement: Supplementary file 2 — Additional file 2: Appendix 2. D-dimer levels and platelet levels where reported for cases of COVID-19 in pregnancy. [file 12884_2021_3568_MOESM2_ESM.docx]

Appendix 2: D-dimer levels and platelet levels where reported for cases of COVID-19 in pregnancy

| Study number (as per Appendix 1) | Number of patients with results given | Normal platelets, normal D-dimer | Normal platelets | Normal D-dimer | Low platelets | High D-dimer | Low platelets, high D-dimer | Normal platelets, high D-dimer |
| --- | --- | --- | --- | --- | --- | --- | --- | --- |
| 2a | 2 |  | 1 |  | 1 |  |  |  |
| 4 | 1 |  |  |  |  |  | 1 |  |
| 15 | 7 |  |  |  |  | 5 | 2 |  |
| 19 | 1 |  |  |  |  | 1 |  |  |
| 20 | 1 |  | 1 |  |  |  |  |  |
| 21 | 1 |  |  |  |  |  |  | 1 |
| 28 | 1 |  | 1 |  |  |  |  |  |
| 53 | 1 |  |  |  |  | 1 |  |  |
| 60 | 1 |  | 1 |  |  |  |  |  |
| 62 | 1 |  | 1 |  |  |  |  |  |
| 65 | 1 |  |  |  | 1 |  |  |  |
| 67 | 7 |  | 5 |  | 2 |  |  |  |
| 87 | 1 |  |  |  |  |  |  | 1 |
| 89 | 1 |  |  |  |  |  |  | 1 |
| 91 | 1 |  |  |  |  | 1 |  |  |
| 98 | 1 |  |  | 1 |  |  |  |  |
| 102 | 7 | 3 | 3 |  |  |  |  | 1 |
| 111 | 1 |  |  |  |  |  | 1 |  |
| 140 | 8 | 3 |  |  |  |  | 5 |  |
| 149 | 8 |  |  |  |  |  |  | 8 |
| 152 | 2 |  | 2 |  |  |  |  |  |
| 153 | 1 |  |  |  | 1 |  |  |  |
| 161 | 54 |  | 54 |  |  |  |  |  |
| COV-PREG-COAG | 2 |  |  |  |  | 1 | 1 |  |
| Total | 112 | 6 | 69 | 1 | 5 | 9 | 10 | 12 |

| Low platelets: as defined by author or <100 |
| --- |
| High D-dimer: as defined by author or >500 ng/ml |
